# Advances in cryo-electron microscopy (cryoEM) and X-Ray for structure-based drug discovery

**DOI:** 10.1063/4.0000952

**Published:** 2025-10-27

**Authors:** Pawel Rubach, Wladek Minor

**Affiliations:** 1 University of Virginia, Charlottesville, VA, USA; 2 Warsaw School of Economics, Warsaw, Mazowieckie, Poland

## Abstract

Macromolecular X-ray crystallography (XRC), nuclear magnetic resonance (NMR), and cryo-electron microscopy (cryoEM) are the primary techniques for determining atomic-level, three-dimensional structures of macromolecules essential for drug discovery. With advancements in artificial intelligence (AI) and cryoEM, the Protein Data Bank (PDB) [1] is solidifying its role as a key resource for 3D macromolecular structures. This position underscores the growing need for enhanced quality metrics and robust validation standards for all experimental structures. This presentation examines recent advancements in cryoEM [2] and X-ray [3], analyzing structure quality metrics (i.a. see Fig.1: Average Q-score vs. CCMASK for cryoEM structures), resolution improvements, ligand and water molecule identification, refinement software, and the identification of duplicate submissions that are not necessary duplicates [4]. Nearly 800 protein crystal structures determined at a resolution of 2.5 Å or better are present in the current PDB release without any water molecules. At the same time, some other depositions exhibit unusually low or high solvent occupancies [5]. We will discuss the role of both X-ray and cryoEM techniques in drug discovery in light of two significant findings: the demonstration that cryoEM can resolve hydrogen atom positions and water networks [6], and the report of an atomic-resolution (1.09 Å) protein structure (8RQB) that reveals double conformations [7].
